# Clinical outcomes of colorectal neoplasm with positive resection margin after endoscopic submucosal dissection

**DOI:** 10.1038/s41598-024-63129-1

**Published:** 2024-05-29

**Authors:** Hyung-Hoon Oh, Je-Seong Kim, Jae-Woong Lim, Chae-June Lim, Young-Eun Seo, Ga-Ram You, Chan-Muk Im, Ki-Hyun Kim, Dong-Hyun Kim, Hyun-Soo Kim, Young-Eun Joo

**Affiliations:** Department of Internal Medicine, Chonnam National University Medical School, 8 Hak-Dong, Dong-ku, Gwangju, 501-757 Republic of Korea

**Keywords:** Colorectal tumor, Endoscopic submucosal dissection, Positive margin, Gastrointestinal cancer, Colonoscopy, Gastrointestinal diseases

## Abstract

A positive resection margin after colorectal endoscopic submucosal dissection (ESD) is associated with an increased risk of recurrence. We aimed to identify the clinical significance of positive resection margins in colorectal neoplasms after ESD. We reviewed 632 patients who had *en bloc* colorectal ESD at two hospitals between 2015 and 2020. The recurrence rates and presence of residual tumor after surgery were evaluated. The rate of additional surgery after ESD and recurrence rate were significantly higher in patients with incomplete resection (n = 75) compared to patients with complete resection (n = 557). When focusing solely on non-invasive lesions, no significant differences in recurrence rates were observed between the groups with complete and incomplete resection (0.2% *vs*. 1.9%, p = 0.057). Among 84 patients with submucosal invasive carcinoma, 39 patients underwent additional surgery due to non-curative resection. Positive vertical margin and lymphovascular invasion were associated with residual tumor. Lymphovascular invasion was associated with lymph node metastasis. However, no residual tumor nor lymph node metastases were found in patients with only one unfavorable histological factor. In conclusion, a positive resection margin in non-invasive colorectal lesions, did not significantly impact the recurrence rate. Also, in T1 colorectal cancer with a positive vertical resection margin, salvage surgery can be considered in selected patients with additional risk factors.

## Introduction

Although colorectal endoscopic submucosal dissection (ESD) is technically challenging, many colorectal neoplasms, including T1 colorectal cancer (CRC), are resected using ESD with advances in colonoscopy performance and the development of endoscopic instruments^1,2^. Using the ESD technique, the tumor can be resected *en bloc*, irrespective of the size and morphology of the tumor. Tumor *en bloc* resection is important since it is associated with lower recurrence than piecemeal resection^3^. Moreover, it allows pathologic evaluation of tumor involvement in the vertical and horizontal margin. In the recent European Society of Gastroenterology (ESGE) guidelines on ESD, a positive horizontal margin is considered local-risk resection, and endoscopic surveillance or endoscopic treatment is recommended. A positive vertical margin is considered a high-risk (non-curative) resection, and additional treatment, such as surgery, is recommended individually^4^.

However, margin involvement of the tumor is not always associated with residual tumors. Normal mucosa and neoplasia are easily distinguishable, and ESD has the advantage of continuous optical control during the precutting of the lateral margin and the dissection of the submucosal layer. Further, a positive resection margin may be caused by cauterization or tangential specimen cutting in the pathology department^5,6^.

Endoscopic resection for local residual/recurrent colorectal tumors is technically difficult as sufficient submucosal injection may not be possible due to submucosal fibrosis. Although recent studies reported that salvage ESD for local/recurrent colorectal tumors is effective and safe, most of the tumors included in this study were resected using the endoscopic mucosal resection (EMR) method, and most of the procedure was performed by experts in large volume centers^7^. For patients with non-curative resected T1 CRC, there is still controversy whether additional surgery is necessary or if surveillance alone is sufficient^8^. Although current guidelines recommend surgery, residual tumor was found in less than 7–18% of patients in previous studies^9,10^. Colorectal surgery can cause significant morbidity and mortality to patients. Therefore, selecting patients who need additional surgery is critical to prevent unnecessary surgical treatment. This study aimed to evaluate the clinical significance of positive resection margin in colorectal neoplasms after ESD.

## Materials and methods

### Study design and patient selection

**Figure 1:**
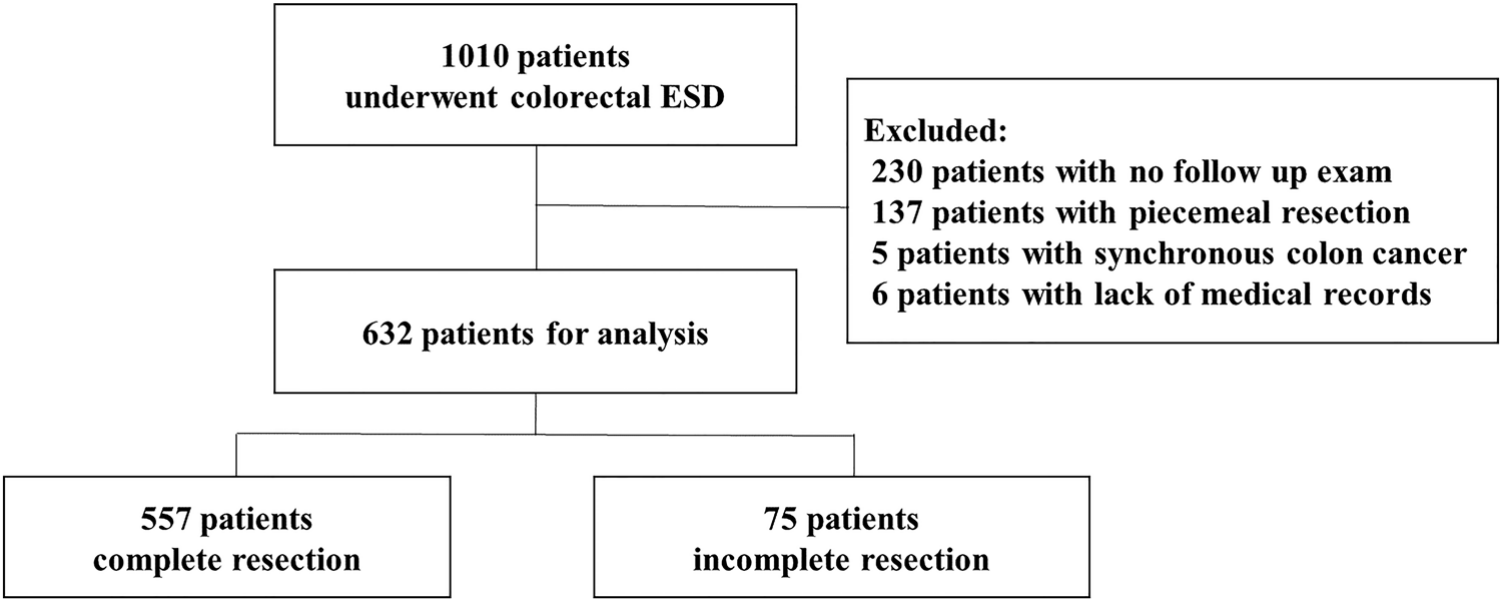
Schematic flow chart of the patients in this study.

This study was a multicenter retrospective cohort study of all the patients submitted to colorectal ESD at two tertiary medical centers (Chonnam National University Hwasun Hospital and Chonnam National University Hospital) located in South Korea between January 2015 and December 2020. Indications for colorectal tumors treated using ESD followed the Korean Journal of Gastroenterology guidelines. (1) Clinically diagnosed intramucosal neoplasm, regardless of cancer or adenomas, or invasive carcinoma with superficial submucosal invasion (SM1; < 1000 µm from muscularis mucosa) using chromoendoscopy or magnification, (2) lesions larger than 20 mm of any macroscopic type, (3) lesions that are unsuitable for endoscopic mucosal resection (EMR), such as recurrence of the lesion previously treated by endoscopic resection. All the colorectal ESD procedures were performed by experienced endoscopists who had performed over 200 gastric ESD procedures. The endoscopes (such as PCF-Q260JI, CF-H260AI, CF-HQ290I, CF-H290I; all from Olympus, Tokyo, Japan) were chosen in accordance with the location and features of the lesions. Indigo carmine dye was used to delineate the margin of the lesions. A mixture of methylene blue and epinephrine diluted with normal saline was injected into the submucosa to lift the lesion. Mucosal incision and submucosal dissection were conducted with careful hemostasis using a needle knife (Dual knife; Olympus, Tokyo, Japan) or insulated-tipped knife (IT knife; Olympus, Tokyo, Japan). Then, coagulation of visible vessels in the artificial ulcer was performed. The patient inclusion criteria were (1) an *en bloc* resection achieved with endoscopically radical resection and (2) at least one follow-up colonoscopy or surgery was performed after ESD. In the present study, of the 1010 patients who underwent colorectal ESD at two hospitals between January 2015 and December 2020, 632 patients fulfilled our inclusion criteria and were analyzed (Fig. 1). Although many neuroendocrine tumors in the rectum were treated using ESD, they were not included in this study. All methods were carried out in accordance with relevant guidelines and regulation. Informed consent was obtained from all subjects and/or their legal guardians before procedure. The study protocol was approved by the ethical review boards of the two participating institutions (Chonnam National University Hwasun Hospital (IRB No. CNUHH-2022-208), Chonnam National University Hospital (IRB No. CNUH-2022-060)).

### Patients, lesions, and ESD characteristics

Patient baseline characteristics were extracted from their medical records. Information on the lesions (morphology, location, size) and ESD procedural data (degree of fibrosis, degree of nonlifting sign) were extracted from standardized endoscopic reports in both hospitals. *En bloc* resection was defined as an excision of the tumor in one piece without fragmentation.

### Histology

Expert gastrointestinal pathologists reviewed all cases. Pathologic diagnoses were reported following the WHO classification guidelines. Complete resection was defined as both the horizontal and vertical margins being free of cancer or dysplastic cells. Incomplete resection was defined when the resection margin had direct contact with either dysplastic or cancer cells or an indeterminate margin. Superficial invasive carcinoma was defined as submucosal invasive carcinoma that invaded less than 1000 µm from the muscularis mucosa. Deep invasive carcinoma included submucosal invasive carcinoma of more than 1000 µm from the muscularis mucosa. Invasive colorectal neoplasm included only submucosal invasive carcinoma. Carcinoma in situ and intra-mucosal carcinoma were categorized as non-invasive colorectal neoplasms. Unfavorable histological factors were considered as follows: (1) positive vertical margin, (2) a submucosal invasion depth > 1000 µm, (3) lymphovascular invasion, (4) poorly differentiated cancer, and (5) high tumor-budding (budding grade ≥ 2).

### Post-resection management

The first surveillance colonoscopy was performed 3–12 months after ESD according to the pathologic diagnosis. A second surveillance colonoscopy was recommended, according to the result of the first surveillance colonoscopy. The recurrence was defined as lesions at the scar or ESD site. Treatment modality and treatment outcome were evaluated in the patients with recurrence. Additional surgical management was recommended for the patients who had endoscopic resection for T1 CRC with any unfavorable histological factors. In the patients who underwent further surgery, details regarding unfavorable histological factors and the presence of residual tumor and lymph node metastasis in the surgical specimen were evaluated.

### Statistical analysis

Categorical variables were presented as absolute (n) and relative frequencies (%). Mean and SD, or median and interquartile range (IQR), were used for continuous variables as appropriate. The Mann–Whitney U test, the Student’s *t*-test, the chi-square test, or the analysis of variance (ANOVA) was used as appropriate. Statistical significance was set at P < 0.05. All data were analyzed using Statistical Package for the Social Sciences, version 27.0 (SPSS Inc., Chicago, IL, USA).

## Results

### Baseline characteristics

Among 632 patients, 557 had complete resections with negative vertical and horizontal resection margins, and 75 patients had incomplete resections. There were no significant differences in age, sex, polyp morphology, presence of central ulceration or depression, location, fibrosis, non-lifting sign, size of the tumor, and procedure time between patients with complete resection and patients with incomplete resection. The median size of the complete resected tumor was 21.5 ± 10.4, and that of the incomplete resected tumor was 23.7 ± 13.9, which was not significantly different. A total of 549 patients were pathologically diagnosed with non-invasive colorectal neoplasms, including adenoma with dysplasia or serrated adenoma, carcinoma in situ and intra-mucosal carcinoma. 84 patients were diagnosed with invasive colorectal neoplasms, including superficial invasive carcinoma and deep invasive carcinoma. A total of 3.6% (18/557) of the patients with complete resection underwent additional surgery, and 31.9% (21/75) of patients with incomplete resection underwent further surgery, which was significantly different. The recurrence rate was 0.4% (2/557) for complete resection and 2.7% (2/75) for incomplete resection, which was significantly different (Table 1). Details of recurrence cases during follow-up are summarized in Table 2. Among four patients with recurrence, two patients had intramucosal carcinoma, and two had superficial submucosal invasive carcinoma (SM1). One patient with a positive horizontal margin after initial resection had recurrence after 71 months and was transferred to another hospital but remains alive. The other patient with positive vertical margin after initial resection had recurrence after 12 months and underwent laparoscopic anterior resection. The pathologic diagnosis was T1aN0, and they are alive.

**Table 1:** Baseline characteristics of enrolled patients.

Patients	Complete resection (n = 557)	Incomplete resection (n = 75)	P value
Age (mean ± SD)	65.7 ± 10.4	64.0 ± 10.8	0.359
Sex			
Male	334 (60.0)	43 (57.3)	0.654
Female	223 (40.0)	32 (42.7)	
Polyp morphology (Paris classification)			
Type I	105 (18.8)	19 (25.3)	0.182
Type II	452 (81.2)	56 (74.4)	
Central ulceration or depression	138 (24.7)	20 (26.7)	0.716
Location			
Cecum	52 (9.3)	6 (8.0)	0.267
Ascending colon	121 (21.9)	14 (18.7)	
Transverse colon	95 (17.0)	9 (12.0)	
Descending colon	24 (4.3)	2 (2.7)	
Sigmoid colon	122 (21.9)	24 (32.0)	
Rectum	143 (25.6)	20 (26.6)	
Fibrosis			
None	348 (62.4)	45 (60.0)	0.726
Mild	141 (25.4)	19 (25.3)	
Severe	68 (12.2)	11 (14.7)	
Non-lifting sign			
Well lifting	389 (69.7)	48 (64.0)	0.496
Mild non-lifting	115 (20.8)	17 (22.7)	
Severe non-lifting	53 (9.5)	10 (13.3)	
Polyp size			
Long axis	21.5 ± 10.4	23.7 ± 13.9	0.179
Short axis	18.4 ± 9.7	19.8 ± 11.2	0.583
Procedure time (minutes, mean ± SD)	33.6 ± 26.9	38.8 ± 33.8	0.133
Pathologic diagnosis			
Hyperplastic polyp	7 (1.3)	0 (0)	
Sessile serrated lesion	16 (2.9)	2 (2.7)	
Low grade dysplasia	202 (36.2)	19 (25.3)	
Sessile serrated lesion with dysplasia	11 (2.0)	1 (1.3)	
Traditional serrated adenoma	3 (0.5)	0 (0)	
High grade dysplasia/carcinoma in situ	135 (24.7)	18 (24.0)	
Intra-mucosal carcinoma	121 (21.7)	13 (17.1)	
Superficial invasive carcinoma (< 1000 µm)	32 (5.7)	1 (1.3)	
Deep-invasive carcinoma (≥ 1000 µm)	30 (5.4)	21 (28.0)	
Margin status			
Positive horizontal margin		43 (57.3)	
Positive vertical margin		17 (22.7)	
Positive both margins		15 (20.0)	
Follow-up months (mean ± SD)	25.5 ± 65.6	26.6 ± 19.9	0.698
Number of follow-up colonoscopies	2.2 ± 1.5	2.1 ± 1.4	0.178
Underwent additional operation	18 (3.6)	21 (31.9)	** < 0.001**
Recurrence	2 (0.4)	2 (2.7)	**0.018**

Significant values are in bold.

**Table 2:** Details of cases with recurrence during follow-up.

Age	Location	Size	Pathologic diagnosis	Margin status	Number of surveillance colonoscopies	Time to recurrence	Treatment of recurrence	Pathologic report	Survival
52	Sigmoid colon	22 × 18 mm	Tubular adenoma with high grade dysplasia and intra-mucosal carcinoma	Horizontal margin positive	5	71 months	Local transferred	Unknown	Alive
47	Cecum	30 × 25 mm	Intra-mucosal carcinoma	R0 resection	4	46 months	Laparoscopic cecectomy	pT1aN0	Alive
73	Recto-sigmoid junction	38 × 34 mm	Submucosal invasive carcinoma (SM1)	R0 resection	5	32 months	Laparoscopic anterior resection	pT3N1	Alive
73	Recto-sigmoid junction	24 × 18 mm	Submucosal invasive carcinoma (SM1)	Vertical margin positive	1	12 months	Laparoscopic. anterior resection	pT1aN0	Alive

### Clinical outcome of patients with non-invasive colorectal neoplasm

Among 632 patients, 549 patients were diagnosed with non-invasive colorectal neoplasms, including hyperplastic polyp, sessile serrated lesion, adenoma with low-grade dysplasia, sessile serrated lesion with dysplasia, traditional serrated adenoma or adenoma with high-grade dysplasia and intra-mucosal carcinoma. A total of 496 patients had complete resections, and 53 patients had incomplete resections. The median long axis of the tumor was significantly shorter in the complete resection group compared to the incomplete resection group (21.7 ± 10.5 *vs.* 25.3 ± 15.1, p = 0.021). However, there was no significant difference in the recurrence rate between two groups with rates of 0.2% *vs.* 1.9% over median follow-up periods of 24.2 months and 23.6 months, respectively (Table 3).

**Table 3:** Characteristics of patients with non-invasive colorectal neoplasm.

Patients ( n = 549)	Complete resection (n = 496)	Incomplete resection (n = 53)	P value
Size (mm, mean ± SD)			
Long axis	21.7 ± 10.5	25.3 ± 15.1	**0.021**
Short axis	18.5 ± 9.6	21.0 ± 12.0	0.083
Final diagnosis, n (%)			
HP	7 (1.4)	0	
SSL	16 (3.2)	2 (3.8)	
LGD	202 (40.7)	19 (35.9)	
SSL with dysplasia	11 (2.2)	1 (1.9)	
TSA	3 (0.6)		
HGD	136 (37.5)	18 (34.0)	
Intra-mucosal carcinoma	121 (24.4)	13 (24.5)	
Margin status			
Positive horizontal margin		48 (90.6)	
Positive vertical margin		4 (7.5)	
Positive both margins		1 (1.9)	
Recurrence	1 (0.2)	1 (1.9)	0.054
Follow-up months	24.2	23.6	0.752

HP, hyperplastic polyp; SSL, sessile serrated lesion; LGD, low grade dysplasia; TSA, traditional serrated adenoma; HGD, high grade dysplasia.

Significant values are in bold.

### Clinical outcome of patients with invasive colorectal neoplasm

84 patients were diagnosed with invasive colorectal neoplasm. Among them, 22 patients had incomplete resections. Patients with deep invasive carcinoma (submucosal invasion depth ≥ 1000 µm) had significantly higher rate of incomplete resections compared to superficial invasive carcinoma (submucosal invasion depth < 1000 µm). Also, patients with incomplete resections had significantly higher rate of operation compared to patients with complete resections (90.1% *vs.* 30.6% respectively, p < 0.001) (Table 4).

**Table 4:** Characteristics of patients with invasive colorectal neoplasm.

Patients (n = 84)	Complete resection (n = 62)	Incomplete resection (n = 22)	P value
Size (mm, mean ± SD)			
Long axis	20.4 ± 10.1	19.7 ± 9.8	0.775
Short axis	17.7 ± 10.5	16.6 ± 8.6	0.663
Final diagnosis, n (%)			
Superficial invasive carcinoma (submucosal invasion depth < 1000 µm)	32 (97.0)	1 (3.0)	**0.001**
Deep-invasive carcinoma (submucosal invasion depth ≥ 1000 µm)	30 (58.8)	21 (41.2)	
Margin status, n (%)			
Positive horizontal margin		3 (13.6)	
Positive vertical margin		12 (54.5)	
Positive both margins		7 (31.8)	
Recurrence, n (%)	1 (1.7)	1 (4.5)	0.454
Underwent operation, n (%)	19 (30.6)	20 (90.1)	**﻿0.001**
Follow-up months	36.2 ± 21.8	32.6 ± 20.7	0.513

Significant values are in bold.

### Histology of patients who underwent additional surgery after ESD

Among 18 patients with complete resection, 15 patients had one unfavorable histology, including more than 1000 µm submucosal invasion, poorly differentiated cancer, and high tumor budding grade, showing no residual tumor in the surgical specimen. However, among 3 patients who had two unfavorable histology, one patient with more than 1000 µm submucosal invasion and lymphovascular invasion had a residual tumor in the surgical specimen.

Among 21 patients with incomplete resection, 4 patients with only vertical margin positive had no residual tumor in the surgical specimen. Moreover, patients with positive horizontal margin and submucosal invasion > 1000 µm showed no residual tumor in the surgical specimen. However, among 12 patients who had positive vertical margins and at least one unfavorable histology (such as a submucosal invasion > 1000 µm, lymphovascular invasion, poorly differentiated cancer, and high tumor budding grade), seven patients had residual tumors in the surgical specimen. Among them, there was two patients with intramural cancer (Tables 5 and 6).

**Table 5:** Pathological parameters and the presence of residual tumor in patients who underwent additional surgery.

Reason for operation	Complete resection n = 18		Incomplete resection n = 21	
	Number of surgery patients	Residual tumor	Number of surgery patients	Residual tumor
Positive vertical margin			4	0
> 1000 µm submucosal invasion	12	0	4	0
Positive vertical margin + other risk factors	0		12^a^	7
> 1000 µm submucosal invasion + other risk factors	3^b^	1		
Poorly differentiated cancer	1	0		
Tumor budding	2	0		
Non-specific^c^	0		1	0

^a^9 patients with more than > 1000 µm submucosal invasion/2 patients with more than > 1000 µm submucosal invasion and poorly differentiated cancer/1 patient with lymphovascular invasion.

^b^2 patients with lymphovascular invasions/1 patient with poorly differentiated cancer.

^c^Preference of the patient for surgical treatment despite having no histological high-risk factors (T1SM1).

**Table 6:** Details of cases with the residual tumors in the surgical specimens.

Age	Location	Tumor size (mm)	Reasons for operation	Method of operation	Pathologic report	Follow-up period
57	Rectum	12 × 10	> 1000 µm submucosal invasion Vertical margin positive	Transanal excision	T1N0	10 months
56	Rectum	12 × 9	> 1000 µm submucosal invasion Vertical margin positive	Laparoscopic LAR	T1N0	42 months
74	Rectum	18 × 8	Lymphovascular invasion > 1000 µm submucosal invasion Vertical margin positive	Laparoscopic LAR	T1N1	59 months
62	Sigmoid colon	20 × 20	> 1000 µm submucosal invasion Vertical margin positive Horizontal margin positive	Laparoscopic AR	T3N1	59 months
71	Sigmoid colon	20 × 10	> 1000 µm submucosal invasion Vertical margin positive	Laparoscopic LAR	T1N0	57 months
71	Sigmoid colon	18 × 15	> 1000 µm submucosal invasion Vertical margin positive	AR	T1N0	20 months
79	Ascending colon	25 × 22	Lymphovascular invasion > 1000 µm submucosal invasion	Laparoscopic right hemicolectomy	T1N1	12 months
73	Descending colon	8 × 7.5	> 1000 µm submucosal invasion Vertical margin positive	Right hemicolectomy	T3N0	10 months

LAR, low-anterior resection; AR anterior resection; ESD, endoscopic-submucosal dissection.

### Additional surgery in patients with non-curative resected colorectal neoplasm

Among 632 patients, 84 were diagnosed with invasive colorectal neoplasm. Among them, 39 patients underwent additional surgery due to unfavorable histologic results after non-curative ESD. First, we analyzed factors associated with residual tumors in the colonic wall. Positive horizontal margin, carcinoma differentiation, depth of invasion, and high tumor-budding grade were unrelated to residual colonic wall tumors. However, positive deep margin and lymphovascular invasion were significantly associated with residual tumors in the colonic wall. Second, we analyzed factors associated with lymph node metastasis. Positive horizontal margin, positive vertical margin, carcinoma differentiation, invasion depth, and tumor-budding grade were not associated with lymph node metastasis. However, presence of lymphovascular invasion was significantly associated with lymph node metastasis. Additionally, there were no residual tumors or lymph node metastasis in 25 patients with only one unfavorable histological factor. However, in 14 patients with more than one unfavorable histological factor, eight (57.1%) patients had residual tumors, and four (28.6%) patients had lymph node metastasis, which was statistically significantly different (p < 0.001 and p = 0.006, respectively) (Table 7).

**Table 7:** Unfavorable histological factors and presence of residual tumor and lymph node metastasis.

	n	Residual tumor			Lymph node metastasis		
		No	Yes	P value	No	Yes	P value
Histopathological analysis							
Horizontal margin				0.732			0.442
Negative horizontal margin	34	27(79.4)	7 (20.6)		31 (91.2)	3 (8.8)	
Positive horizontal margin	5	4 (80.0)	1 (20.0)		4 (80.0)	1 (20.0)	
Vertical margin				**0.003**			0.145
Negative vertical margin	23	22 (95.7)	1 (4.3)		22 (95.7)	1 (4.3)	
Positive vertical margin	16	9 (56.3)	7 (43.8)		13 (81.3)	3 (18.8)	
Lymphovascular invasion				**0.039**			**0.001**
Present	36	30 (83.3)	6 (16.7)		34 (94.4)	2 (5.6)	
Absent	3	1 (33.3)	2 (66.7)		1 (33.3)	2 (66.7)	
Differentiation				0.284			0.475
Well/moderate	35	27 (77.1)	8 (22.9)		31 (88.6)	4 (11.4)	
Poor	4	4 (100.0)	0 (0)		4 (100.0)	0 (0)	
Depth of invasion				0.107			0.284
Less than 1000 µm	8	8 (100.0)	0 (0)		8 (100.0)	0 (0)	
More than 1000 µm	31	23 (74.2)	8 (25.8)		27 (87.1)	4 (12.9)	
Tumor-budding grade				0.461			0.624
Grade 1	37	29 (78.4)	8 (21.6)		33 (89.2)	4 (10.8)	
Grade 2/3	2	2 (100.0)	0 (0)		2 (100.0)	0 (0)	
Number of unfavorable histological factors^a^				** < 0.001**			**0.006**
One	25	25 (100.0)	0 (0)		25 (100.0)	0 (0)	
More than one	14	6 (42.9)	8 (57.1)		10 (71.4)	4 (28.6)	

^a^Unfavorable histological factors include (1) positive vertical margin, (2) a submucosal invasion depth > 1000 µm, (3) lymphovascular invasion, (4) poorly differentiated cancer, and (5) high tumor-budding grade.

Significant values are in bold.

## Discussion

In this multicenter study, including 1010 cases of colorectal ESD, 632 *en bloc* colorectal ESD patients were enrolled. We excluded piecemeal resected cases because the margin status could not be assessed thoroughly. *En bloc* resection is essential as the recurrence rate after post-R0 resection is reported to be 2.0% (95% CI, 1.3%–3.0%) which is close to zero^11^. Our study showed promising results following colorectal ESD if the lesion was resected *en bloc*; the recurrence rate was only 0.4% for complete resection and 2.7% for incompletely resected colorectal ESD during the mean follow-up period of 25.5 ± 65.6 months and 26.6 ± 19.9 months respectively. Furthermore, when we sub-analyzed only non-invasive lesions, although there was one case of recurrence in each groups, there were no significant difference in recurrence rates in the completely or incompletely resected lesions with a median follow-up of 24 months. Haasnoot et al. reported that with successful *en bloc* ESD for non-invasive colorectal neoplasm, the risk of local recurrence was only 2.1% in positive horizontal margins with no significant risk increase. Unlike the EMR method, ESD allows for continuous visual control during the lateral incision in the procedure. Colorectal neoplasms are easily distinguishable from normal mucosa. Thus, a low local recurrence is expected even if the pathologic diagnosis confirms a positive resection margin. A similar result was reported by Lee et al., where the 5-year cumulative recurrence rate was not significantly different between the R0 group and positive resection margin group. Furthermore, 32.2% of the positive horizontal margin cases were confirmed false positives after the specimen was histologically reassessed. The authors suggested three reasons for these false positive results. First, the horizontal margin may be positive due to the sectioning line parallel to the horizontal margin. Second, as the specimen after ESD is very thin, the fragile edge may be distorted during the embedding procedure. Third, as the tissue is cut by electrocautery, a coagulation artifact is made at the lateral margin, which may cause misdiagnoses by pathologists^12^. Additionally, the definition of positive resection margin varies between groups, with some groups defining R1 resection as direct contact between the tumor and the resection margin and others defining R1 resection as less than 1 mm distance between the tumor and the resection margin^13–16^. Taken together, a positive resection margin after *en bloc* ESD is considered safe, especially for non-invasive colorectal neoplasm.

This study included 218 colorectal neoplasms with adenocarcinoma. Among them, 39 patients underwent additional operations due to non-curative resection. The remaining 179 patients underwent surveillance for an average of 37.2 months with an average of 3.0 colonoscopies. According to the large meta-analysis, 96% of residual tumors are found in the first surveillance colonoscopy 6 months after resection and 98% in one year^17^. Therefore, the follow-up duration was adequate for detecting residual tumors. In this study, four patients had recurrence during follow-up, three patients underwent surgery, and one patient was transferred to another hospital per the patient's request. All of them had curative surgery and are under regular follow-ups. Shin et al. analyzed 265 patients who had endoscopic resection for T1 CRC with a positive resection margin. A total of 213 patients underwent additional surgery, and only 13 patients (6.1%) had residual tumors. Further, 52 patients did not undergo extra surgery; only four had recurrence and salvage surgery with good treatment outcomes^9^. In the current ESGE guidelines, positive vertical margin, a submucosal invasion depth > 1000 µm, lymphovascular invasion, poorly differentiated cancer, and high tumor budding in the pathologic exam are considered high-risk (non-curative) resection and additional treatment, such as surgery, is recommended on an individual basis^4^. Although surgery after colorectal ESD is considered effective and safe^18,19^, can cause significant morbidity and mortality to patients^20^. Therefore, selecting patients who need additional surgery is crucial.

In this study, we analyzed 39 patients who underwent additional surgery due to unfavorable histological factors. The residual tumor was associated with a positive deep margin and the presence of lymphovascular invasion. There are only a few studies on residual tumors after ESD. João Santos-Antunes et al. reported that piecemeal resection, poorly differentiated tumors, and positive vertical margin were associated with residual tumors at the ESD site after analyzing 135 T1 CRCs using non-curative ESD^10,21^. Although our study did not include piecemeal resected cases, the result was inconsistent with previous studies. Considering lymph node metastasis, only the presence of lymphovascular invasion was associated with lymph node metastasis in this study. However, due to the inclusion of only four cases with lymph node metastasis in this study, caution is needed when interpreting the results. João Santos-Antunes et al. reported that poorly differentiated cancer was also associated with lymph node metastasis^10^. A meta-analysis of the histopathologic factors related to lymph node metastasis and recurrence in T1 CRC revealed that a submucosal invasion depth of more than 1000 µm, lymphovascular invasion, poorly differentiated tumor, and high tumor-budding grade were found to be significantly associated with lymph node metastasis and recurrence^22,23^. However, little is known about the clinical significance of a single risk factor or a combination in lymph node metastasis or recurrence. In this study, patients with only one unfavorable histological factor had no residual tumor or lymph node metastasis. However, in patients with more than one unfavorable histological factor, 8 out of 14 (57.1%) patients had residual tumors with two patients with intramural cancer, and 4 (28.6%) had lymph node metastases, which was statistically significant. Also, recent large-volume study reported that there was no significant difference in tumor recurrence and disease specific survival rate between patients with non-curative resected T1 CRC who underwent additional surgery and those who were managed through surveillance alone. With this results, they concluded follow-up may be considered in patients at high risk of surgery^24^. Although the sample size was small and caution is needed in interpreting the results, our results suggest that patients with only one risk factor and has high risk of surgery may consider surveillance instead of surgery. However, patients with more than one risk factor after non-curative resection for T1 CRC need to consider additional surgery. There has been an effort to predict the risk of residual tumors or lymph node metastases using computer-aided models or artificial intelligence^25,26^. However, these studies did not evaluate the endoscopically removed CRC but only the surgically removed early CRCs.

This study has strengths and limitations. The main limitation is that selection bias was inevitable owing to the retrospective nature of this study, and the follow-up period for certain patients may be too short. In addition, the number of patients with recurrence was relatively small. However, the main strength of this study is that it involved a large number of colorectal ESD cases with a large number of invasive colorectal neoplasms.

In conclusion, our study demonstrated that a positive resection margin in non-invasive colorectal lesions, including those classified as Tis, did not significantly impact the recurrence rate, suggesting a potential for conservative management in select patients with well-defined criteria. In T1 colorectal cancers with a positive vertical resection margin, salvage surgery can be considered in selected patients with additional risk factors such as submucosal invasion depth > 1000 µm, lymphovascular invasion, poorly differentiated tumor, and high tumor-budding grade. Patients with only one risk factor may delay the operation and consider CT and an endoscopic follow-up.

## Data Availability

All data generated or analysed during this study are included in this published article.
